# Drivers of diversity and community structure of bees in an agroecological region of Zimbabwe

**DOI:** 10.1002/ece3.7492

**Published:** 2021-05-01

**Authors:** Gugulethu Tarakini, Abel Chemura, Tawanda Tarakini, Robert Musundire

**Affiliations:** ^1^ Department of Crop science and Post‐Harvest Technology Chinhoyi University of Technology Chinhoyi Zimbabwe; ^2^ Research and Education for Sustainable Actions Chinhoyi Zimbabwe; ^3^ Department of Environmental Science & Technology Chinhoyi University of Technology Chinhoyi Zimbabwe; ^4^ Potsdam Institute for Climate Impact Research Member of the Leibniz Association Potsdam Germany; ^5^ Department of Wildlife Ecology and Conservation Chinhoyi University of Technology Chinhoyi Zimbabwe

**Keywords:** bees, diversity, forage, land use, pollinator conservation, weather, Zimbabwe

## Abstract

1. Worldwide bees provide an important ecosystem service of plant pollination. Climate change and land‐use changes are among drivers threatening bee survival with mounting evidence of species decline and extinction. In developing countries, rural areas constitute a significant proportion of the country's land, but information is lacking on how different habitat types and weather patterns in these areas influence bee populations.
2. This study investigated how weather variables and habitat‐related factors influence the abundance, diversity, and distribution of bees across seasons in a farming rural area of Zimbabwe. Bees were systematically sampled in five habitat types (natural woodlots, pastures, homesteads, fields, and gardens) recording ground cover, grass height, flower abundance and types, tree abundance and recorded elevation, temperature, light intensity, wind speed, wind direction, and humidity. Zero‐inflated models, censored regression models, and PCAs were used to understand the influence of explanatory variables on bee community composition, abundance, and diversity.
3. Bee abundance was positively influenced by the number of plant species in flower (*p* < .0001). Bee abundance increased with increasing temperatures up to 28.5°C, but beyond this, temperature was negatively associated with bee abundance. Increasing wind speeds marginally decreased probability of finding bees.
4. Bee diversity was highest in fields, homesteads, and natural woodlots compared with other habitats, and the contributions of the genus *Apis* were disproportionately high across all habitats. The genus *Megachile* was mostly associated with homesteads, while *Nomia* was associated with grasslands.
5. Synthesis and applications. Our study suggests that some bee species could become more proliferous in certain habitats, thus compromising diversity and consequently ecosystem services. These results highlight the importance of setting aside bee‐friendly habitats that can be refuge sites for species susceptible to land‐use changes.

## Graphical abstract

Habitat types had differential impacts on bee populations and communities highlighting the need to set aside natural habitats to act as refuge sites for species that cannot tolerant some land‐use changes.

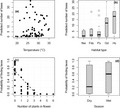

## 1 INTRODUCTION

There is compelling evidence in biodiversity‐related studies that species population trends are declining, but such studies have been biased toward terrestrial and aquatic vertebrates (Ceballos et al., 2017). It has only been recent that insects have been assessed and similar trends of biodiversity loss are being reported (Sánchez‐Bayo & Wyckhuys, 2019) with evidence of dwindling population sizes and range shrinkages. Information on insects is still limited and focused on their category of threat according to the categories of the IUCN Red List (Azam et al., 2016; Ceballos et al., 2017). As such, very little is understood to date on their population status and major factors driving their abundance, diversity, and distribution in order to guide conservation activities.

Previous studies have increasingly recognized the ecological and economic value of insect pollinators with global economic value of wild and managed pollination services estimated at US$215 billion in Southern Africa (Hein, 2009). With the continuing increase in the cultivation of pollinator‐dependent crops, demand for insect pollinators has risen threefold since 1961 (Aizen & Harder, 2009); hence, urgent information is required for their conservation.

Many species of insects are considered to be plant pollinators including beetles, wasps, flies, moths, butterflies, and bees (Rosas‐Guerrero et al., 2014). Bees in particular are considered to be among the most important pollinators in many ecosystems with entomophilous plants (Fleming & Muchhala, 2008; Kevan et al., 1990). *Apis mellifera,* for example, is important for pollination of large monoculture fields (Klein et al., 2003; Klein et al., 2006). Several studies have already documented poor yields in some areas attributable to decline in pollination services (Garibaldi et al., 2016), hence threatening both food security and economic development in these countries. These challenges may greatly impact developing countries’ economies, which are agriculture‐based with over 2 billion of the population being smallholder farmers (Lowder et al., 2016). Thus information on drivers of bee abundance, diversity, and distribution is lacking to guide the development of bee‐friendly habitats and management systems.

Habitat change is one of the major drivers of bee losses as it is normally characterized by habitat loss with shifts in the vegetation composition (i.e., trees, flowers, grasses, herbs, ground cover), which are critical bee habitat and forage requirements (Decourtye et al., 2010). In Zimbabwe, land‐use changes altered habitats resulting in rapid forest loss of 312 thousand hectares per year during 2010–2015 period (MacDicken, 2015). The impacts of the resulting habitat changes are nonrandom, and some species may proliferate in the new environment, yet susceptible species may be lost or experience range contraction (Cely‐Santos & Philpott, 2019). For instance, urbanization has been shown to be more destructive on bee species that nest underground compared with those that build nests in cavities (Lázaro & Tur‐Tur, 2018). Activities that promote such species homogenization may ultimately impact negatively the stability and functioning of an ecosystem considering specialization exhibited by plant–pollinator interactions (Cely‐Santos & Philpott, 2019).

The majority of the human population in Zimbabwe is based in the rural areas (Shumba, 2001), which is an important sector that requires attention in bee conservation as the main livelihood source is agriculture (Tarakini et al., 2020). Rural areas in Zimbabwe are dominated by the following habitats: natural woodlots, pastures, all year round vegetable gardens, fields, and homesteads (Sibanda, 1990). Given the contrasting nature of these habitat types in terms of the magnitude and type of disturbances, it is vital to understand bee community population dynamics in these habitats. Understanding the drivers of bee populations and communities allows for the identification and/or development of specific management options that can be applied to conserve not only individual species but also entire assemblages (Murray et al., 2009).

Suitable abiotic conditions (local climatic conditions and topography) are also important for the survival of bees. Previous researches have shown that weather elements such as temperature, light intensity, wind speed, and rainfall may alter bee species behavior (Hennessy et al., 2020; Rajkhowa & Deka, 2013) and hence ultimately influencing bee abundance, diversity, and distribution. Different bee species have different weather preferences and also take less than a minute to react to weather changes (Riessberger & Crailsheim, 1997). However, most studies on weather effects on bees are biased toward laboratory investigations (Cooper et al., 1985; Hennessy et al., 2020), which cannot assess the synergistic impacts of habitat type and weather parameters, yet habitats due to their unique structure and composition may buffer or increase adverse effects of weather conditions. This information is important in guiding management decisions in the development of area‐specific conservation efforts of greater impact. For example, Papanikolaou et al. (2017) found that semi‐natural habitats mitigated the effects of temperature rise on wild bees within agricultural areas. Furthermore, most assessments on weather have been conducted on honey bees (*Apis mellifera*) with paucity of information on other species especially solitary bees. An understanding of the response of different bee species to weather parameters is important in understanding the level of vulnerability of bee species to climate change (Hodkinson, 2005) and to guide the development of climate change‐proof bee habitats (Murray et al., 2009). Some bee species, for example, have responded to climate change by changing geographic distribution and the plant species they interact with (Schweiger et al., 2008).

Seasonality is another crucial factor influencing bee abundance, diversity, and distribution (Abrahamczyk et al., 2011) with generally more diverse bees in warm and wet months when compared to cold (or hot) and dry periods. (Michener, 1979, 2007). Besides weather differences across seasons, food availability also varies with seasons ultimately influencing local abundance of bees as highlighted by Abrahamczyk et al. (2011) and Gurr (1957). However, little is known about the effect of seasonal changes in bee abundance and diversity in a given locality, yet the information can be important in assessing species risk to seasonal management activities such as applications of pesticides and other agrochemicals in agroecosystems.

In contributing to the development of management options for bee assemblages, this study therefore aimed at exploring potential factors affecting bee communities in various habitats of Zvimba District in Zimbabwe. The study hypothesized that (a) maximal bee diversity and abundance will be recorded at certain ranges of weather parameters, and deviations from such ranges would have detrimental effects; (b) the effect of weather elements on diversity and abundance of bee genera is not uniform across various habitat types, (c) increase in number of plant species in flower, number of trees, grass cover, and ground cover would positively influence bee abundance and diversity; (d) natural woodlots would have a significantly higher bee abundance and bee genera diversity compared with other habitat types; and (e) the wet season would have significantly higher bee diversity and abundance compared with the dry season since the latter generally have scarce floral resources (Williams & Middleton, 2008). Findings from this study bear significance in management and decision‐making purposes such as identifying vulnerable habitats for focused conservation and development of bee conservation strategies.

## 2 MATERIALS AND METHODS

### 2.1 Study area

**FIGURE 1:**
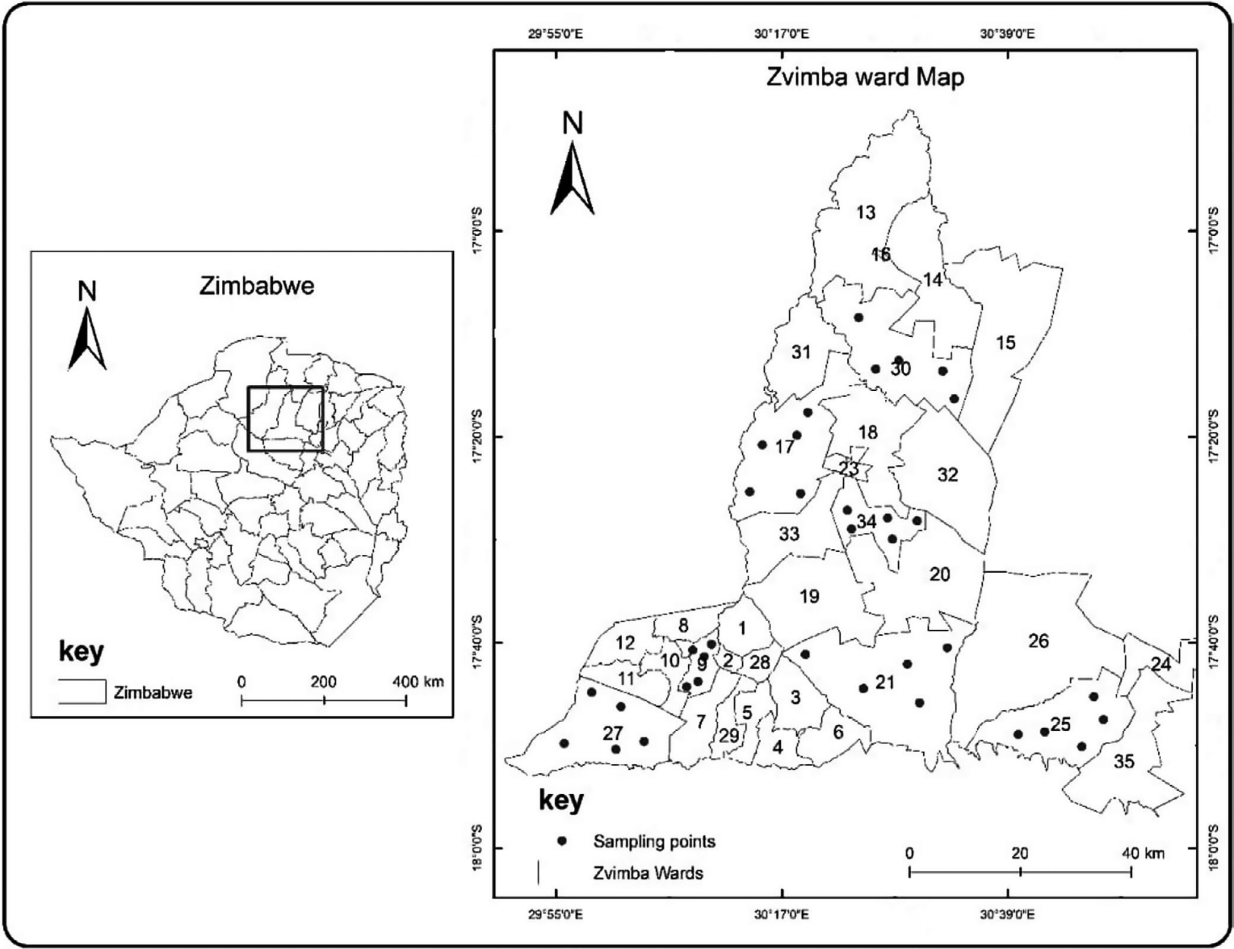
Map showing Zvimba District and the sampling points

The study was carried out in Zvimba District, Mashonaland West Province of Zimbabwe (Figure 1). The district lies in agroecological region 2 (areas of intensive crop, beef, and dairy production, which has significant contribution to Zimbabwe's food security), and it has average annual temperature ranging from 15 to 24°C and mean annual rainfall ranging from 750 to 1,000 mm. For administrative purposes, the district is divided into 35 wards (a section of a district with designated agricultural extension officers and other government officials who are responsible for farming activities and central governance operations) as outlined by the Zimbabwe National Statistics Agency (2012). Land in the wards is constituted by natural woodlots, homestead areas, fields, vegetable gardens, and pastures, which are dominant habitat types in most rural areas found in the country. The main activities in these habitats are summarized in Table 1. The majority of farmers in the district own between five to ten acres of cropland. Crops grown in rural areas’ croplands are mostly cereals, particularly maize (*Zea mays*), which is wind‐pollinated. In the gardens, insect‐pollinated vegetables such as *Brassica* species, tomatoes *Solanum lycopersicum*, onions *Allium* species, and butternut *Cucurbita* species are grown and form important part of the diet and source of nutrition (Tarakini et al., 2020).

**TABLE 1:** Description of dominant habitat types found in the rural areas of Zvimba District, Zimbabwe

Habitat type	Characteristics and activities
Natural woodlots	Constitute indigenous trees, grasses, and herbs. They are normally used for harvesting firewood and other nontimber forest products such as medicine and fruits
Pastures	Dominated by grass (*Hyparrhenia* species). Cattle are driven to these areas to prevent them from grazing inside fields. Grasses are harvested for construction or other household purposes
Fields	Dry season, land left fallow, weeds growing inside. Wet season, fields are cleared of weeds, maize are mainly planted although some crops such as beans, butternuts, sweet potatoes, peanuts, groundnuts, and okra, and cowpeas can be intercropped at rates that will not negatively affect maize production. Pesticides are used to control pests and diseases
Gardens	Planting is all year round. Major activity is cultivation of vegetables; land is cleared, weeded, and cultivated. Planting beds are formed, and vegetables are sown. Pesticides are used to control pests and diseases. Sited close to water sources for easy watering
Homesteads	Have houses, kraals by the entrance to the homestead, trees grown around the homestead (mainly fruit), garbage pit, and a dishwashing stands

### 2.2 Sampling method

**FIGURE 2:**
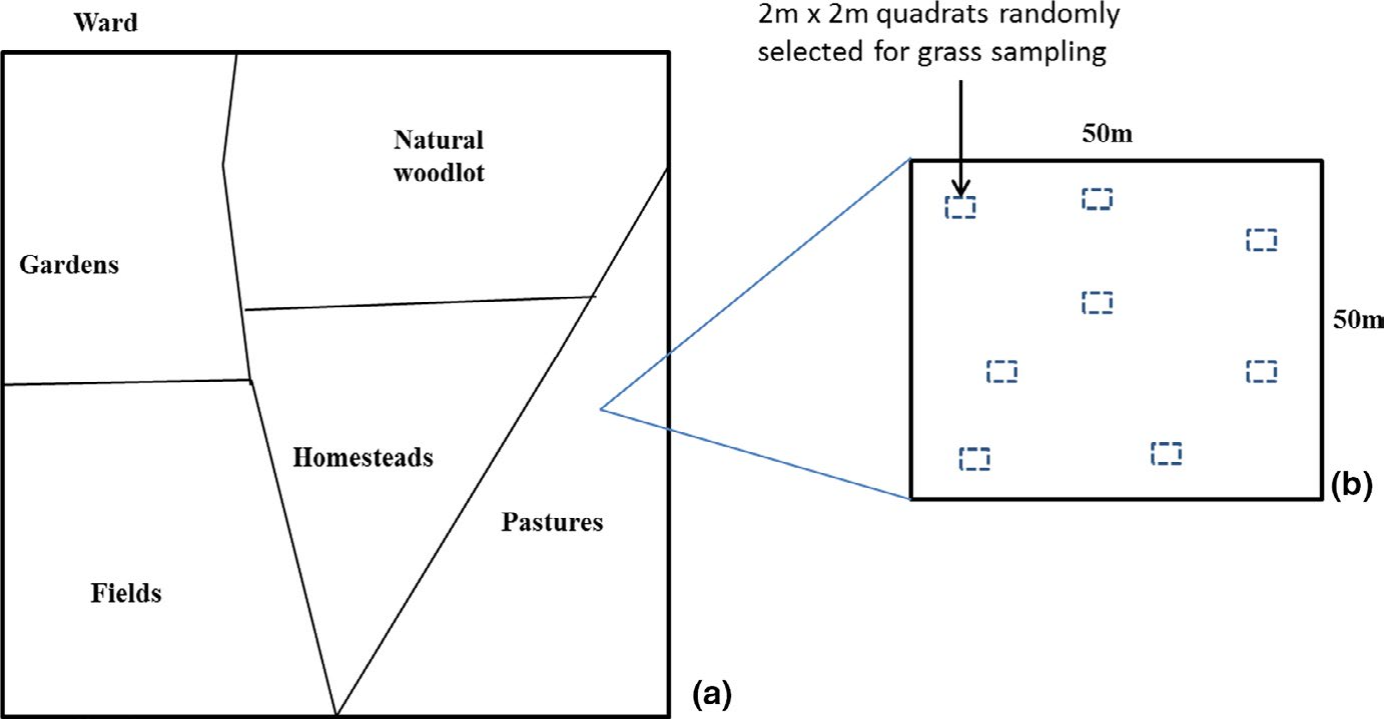
Diagram of the sampling framework of the study (a) shows ward with the different habitat types (b) 50m × 50m plot randomly fixed in each habitat type in the ward to sample bees, trees, and flowers assesses ground cover. Grass cover and grass height were assessed in the 2m x 2m quadrats

Seven wards were randomly selected using Google earth images to avoid bias to specific areas. Five habitat types—(natural woodlots, pastures, homestead, fields, and gardens) with a minimum distance of 2km apart (average foraging distance of bees) (Motzke et al., 2016) (Figure 2)—were randomly selected in each ward. Each habitat type had a minimum size of 2 km^2^, except for gardens that were mostly restricted along rivers or streams. The selected points in each habitat type are hereinafter referred to as sites. Thus, a total of 35 sites in the whole study area were sampled.

### 2.3 Experimental design and vegetation assessments

A 50 × 50 m plot was established on each site to survey bees. The total number of woody plants (tree abundance) and the number of different plant species that were flowering at the time of sampling (hereinafter number of plants in flower) were recorded for each plot. For the plants that were in flower, the number of flowers was enumerated and recorded at each site. In the cases in which a site had large trees that had numerous flowers, an open‐sided wire cube with sides measuring 50 cm was placed on a strategic portion of the canopy and the flowers in the cube were counted. The total number of flowers for such trees was then an extrapolation of approximate number of cubes that could fit in the canopy multiplied by the number of flowers counted in the cube. Eight, 2 × 2 m plots were established within the 50 × 50 m plot to visually estimate ground cover (percentage of ground/soil covered by organic matter), grass cover (percentage of ground covered by grass), and average grass height. A handheld Global Positioning System receiver was used to mark and record the GPS coordinates and elevation of each site.

### 2.4 Bee sampling

Bee sampling was carried out twice in each habitat type (the same 50 × 50 m plots that were used to assess vegetation attributes). The first sampling was carried out in the dry season (July–November 2019) for 35 days and the second time in the wet season (January–March 2020) for 35 days as well. In each season, sampling was conducted on each site for one day covering three time sessions as follows: morning (between 0700 and 0800 hr); early afternoon (1300 and 1400 hr); and late afternoon (1500 and 1600 hr). These different time sessions were used to cater for differences in time of activity for different species (Brunet et al., 2019). Bee sampling was done by a straight walk along 4 transects (1 × 50 m) equidistant from each other within the 50 × 50 m plot for 20 min, and sweep nets were used to capture bees that were flying between 0 and 3 m from the ground, and those that were perching on flowers. Collected bees were pinned and preserved by drying for later identification in the Chinhoyi University of Technology Post‐Harvest Laboratory. The book *Bee Genera and Subgenera of sub‐Saharan Africa* by Eardley et al. (2010) was used to identify bees to genus level. Bees that were flying along the 1 × 50 m transect line but could not be captured with sweep nets were also counted and recorded. A handheld WM‐4 ambient weather meter (manufacturer—Ambient Weather, Chandler, Arizona, USA) was used to record the temperature, humidity, wind speed, and wind direction during each session. Light intensity was measured using the URCERI handheld digital illuminance meter (model number: 4332004118; manufacturer: URCERI, Kansas City Missouri, USA).

### 2.5 Data processing and analysis

The total number of bees observed per site (bee abundance) was derived by adding the numbers of bees that were netted and those that were observed flying in the vicinity of sampling plots. For each site and each season, the vegan package was used to compute the genus Shannon–Weiner diversity index (this was chosen as it accounts for both abundance and evenness of the bee genera present).

A Shapiro–Wilk test was used to check whether bee abundance and Shannon diversity variables were significantly different from normal. The data set obtained had the following explanatory variables: habitat types, season (dry and wet), number of plants in flower, flower abundance, percentage of grass cover, grass height, percentage of ground cover, tree abundance, and weather elements (light intensity, temperature, wind speed, wind direction, humidity). To help in reducing co‐linearity among explanatory variables, all numeric variables were checked for their conformity to normality assumptions using the Shapiro–Wilk test. For the explanatory variables that confirmed normality, Pearson's correlation tests were conducted on groups of ecologically related variables. The first group had weather element variables. The second group consisted of woody vegetation variables (tree abundance, number of different plant species, and ground cover percentage). The third group had herbaceous vegetation variables (i.e., average grass height and percentage grass cover). The fourth group had variables related to flowers (i.e., number of species in flower and flower abundance). In cases, where variables were correlated in each group, one variable was selected for further analysis. This process retained five variables; four of them were related to vegetation and flowers (number of plants in flower, flower abundance, grass cover, ground cover) and two weather parameters (temperature and wind speed). Tree abundance and elevation were not following normality assumptions; thus, they were also included in further analysis. Due to the absence of species in some sites, the bee abundance variable had a lot of zeros; hence, the zero‐inflated models for count data were conducted (using the *GLMMadaptive* package by Rizopoulos (2019)) to test whether the explanatory variables (and all possible 2‐ or 3‐way interactions) influenced bee abundance. Zero‐inflated models are able to incorporate overdispersion and excess zeros in data, and perform analysis in a two‐stage format: (a) a binomial regression that considers presence/absence and (b) a generalized linear model when count is greater than one (Zeileis & Jackman, 2008). There was evidence of quadratic effects in temperature; thus, it was included in the model as a quadratic term.

To assess the relationship between bee diversity and explanatory variables, a censored regression model was used through the *censReg* package (Henningsen, 2010) as it mitigates the problem of zero‐inflated data for continuous response variables (it was not possible to use zero‐inflated model since the Shannon diversity index was not count data). The site identity nested in ward was included in both the zero‐inflated and censored regression models as random variables. Also, for both the zero‐inflated and censored regression models, model selection was done using the backward elimination process, and the best model was selected on the basis of having the lowest Akaike value (AIC). The estimate, standard error, *z* value, and *p* values were reported for the chosen model.

Finally, to investigate the association of bee genera with the explanatory variables, a dataset with computed means for the vegetation and weather parameters was created. To this dataset, the total number of bees belonging to each genus per habitat category, and season were added. To illustrate the effect of the various habitats on each genus, the category that had the highest frequency of that genus was considered. In the cases where habitat categories had the same proportion, the one with the highest frequency for that particular bee genus was considered. A principal component analysis (PCA) was then conducted, and triplots were used to illustrate the result. All analyses were conducted using the R statistical package (R Development Core Team, 2020).

## 3 RESULTS

### 3.1 Descriptive statistics of results

Mean relative humidity during the wet and dry seasons was 61% and 27%, respectively. The sampling sites had altitudes ranging from 1,041 to 1,397 m.a.s.l. and wind speeds ranging from 0 to 6.8 knots.

#### 3.1.1 Environmental factors

The means of ground cover, tree abundance, number of trees in flower, and temperature across habitat types and seasons are presented in Table 2. Average percentage ground cover was highest in gardens followed by fields and least in homesteads. Tree abundance was highest in natural woodlots followed by homesteads and least in pastures. The highest number of plant species in flower was recorded in natural woodlots and least in gardens. Temperatures were highest in homesteads and lowest in pastures (Table 2).

**TABLE 2:** The mean and standard deviation of vegetation and climatic factors recorded across habitat types during dry season of August 2019 and wet seasons of January 2020 in Zvimba District of Zimbabwe

Aspect	Level	Ground cover (%)	Tree abundance	Number of plant species in flower	Temperature (°C)
Habitat	Fields	39.4 ± 21.8	1.6 ± 0.4	1.5 ± 1	28.4 ± 4.5
	Garden	41.8 ± 20.9	1.4 ± 0.7	1.3 ± 1.2	27.4 ± 4.7
	Pastures	33.1 ± 29.3	0 ± 0	1.5 ± 0.9	26.9 ± 2.9
	Homestead	17 ± 8.6	10.9 ± 6.4	1.4 ± 1.2	29 ± 2.7
	Natural	28.6 ± 21.8	14.4 ± 6.4	2 ± 1.9	27.7 ± 2.9
Season	Dry	28.8 ± 24.9	‐	1.1 ± 0.9	28.6 ± 3.9
	Wet	31.9 ± 25.1	‐	1.7 ± 1.2	27.1 ± 3.3

#### 3.1.2 Effect of season on environmental factors

Overall, there was a higher average percentage ground cover during the wet season compared with dry season. Temperatures were lower during wet in comparison with dry season (Table 2). Flower abundance was also higher during wet (222 ± 109) when compared to the dry season (209 ± 98).

**FIGURE 3:**
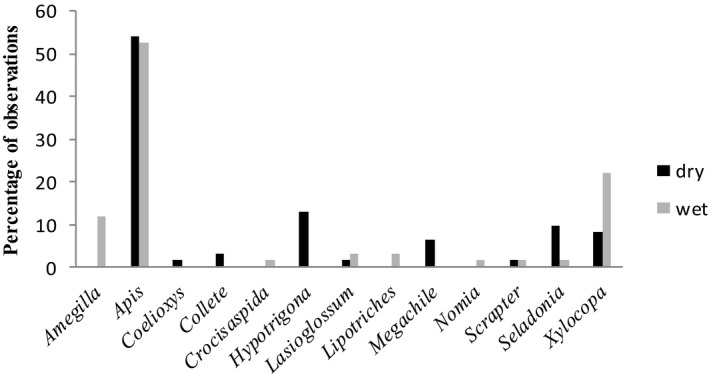
Species observations across dry season of August 2019 and wet seasons of January 2020 in seven sampling sites of Zvimba District in Zimbabwe

A total of 2,961 bees belonging to 13 genera were recorded from the study area. The genus that had the highest observations was *Apis* (53%) (Figure 3) contributing 73% of observations recorded in fields, 53% in gardens, 80% pastures, 68% homesteads, and 23% in natural woodlots. The genera *Coelioxys, Hypotrigona, Megachile,* and *Collete* were only recorded in the dry season, while *Amegilla, Crocisaspida, Lipotriches,* and *Nomia* were recorded only during the wet season.

### 3.2 Influence of environmental and land‐use‐mediated factors on the abundance and diversity of bees

**FIGURE 4:**
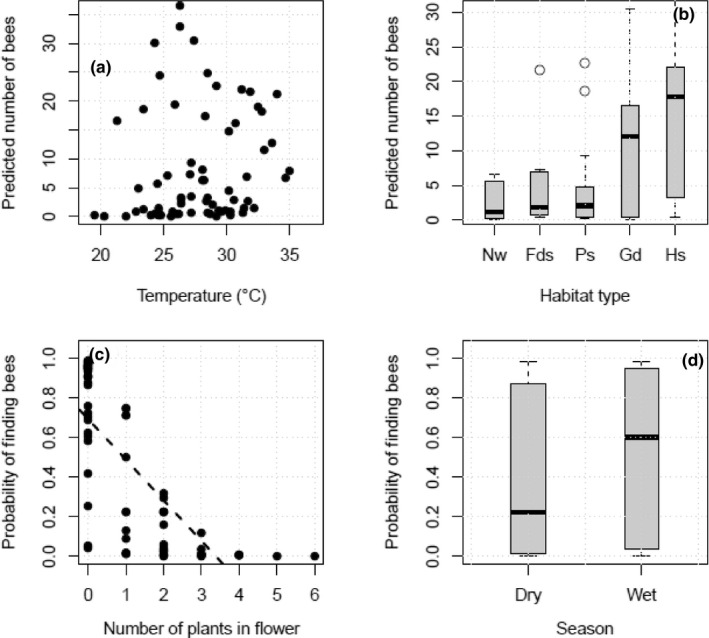
A and B shows influence of temperature and habitat type respectively on number of bees observed. C and D show the probability of finding bees as influenced by number of plants in flower and seasonality respectively in seven sampling sites of Zvimba District in Zimbabwe (August 2019–January 2020). Key: (b) Nw—natural woodlots, Fds—fields, Ps—pastures, Gd—garden, Hs—homesteads (THIS FIGURE IS TO BE USED FOR THE GRAPHICAL TABLE OF CONTENTS AND PUBLICATION COVER.)

The best model describing the abundance of bees retained number of plant species in flower, temperature, and habitat type as explanatory variables (Table 3). The abundance of bees increased with an increase in the number of plant species in flower (*β = *.319, SE = 0.070, *p* < .0001). There was a quadratic effect of temperature on bee abundance. When temperatures were lower than 28.5°C, bee abundance significantly increased with increase in temperature (*β* = 3.521, SE = 1.234, *p* = .004). However, for temperatures above 28.5^0^C, there was a significant decrease (*β *= −2.022, SE = 0.783, *p* = .009) as illustrated in Figure 4a. There were significantly different bee abundances across habitat types (*p* < .0001), with natural woodlots having the least and homesteads the most number of bees as illustrated in Figure 4b, Table 3.

**TABLE 3:** Zero‐inflated model showing the influence of human and environmental factors on a) bee abundance and b) probability of finding bees in seven sampling sites of Zvimba District Zimbabwe (August 2019–January 2020)

Predictors	*Β*	SE		z value	P‐value
**(a) Count model coefficients**					
Intercept	1.827	0.426		4.292	< 0.0001
Number of plants in flower	0.319	0.070		4.594	< 0.0001
Poly (temperature, 2)1	3.521	1.234		2.851	0.004
Poly (temperature, 2)2	−2.022	0.783		−2.582	0.009
Habitat type (garden)	0.369	0.535		0.690	0.490
Habitat type (pastures)	0.211	0.612		0.346	0.730
Habitat type (homestead)	0.397	0.513		0.774	0.439
Habitat type (natural)	−1.720	0.553		−3.112	0.002
**(b) Zero‐part coefficients**					
Intercept	0.234	0.898	0.261		0.794
Wet season	2.521	1.075	2.345		0.019
Number of plants in flower	−2.052	0.565	−3.632		0.0002
Tree abundance	−0.208	0.107	−1.941		0.052
Wind speed	−0.913	0.526	1.737		0.049

The probability of observing bees decreased with increasing number of plant species in flower (*β *= −2.052, SE = 0.565, *p* = .0002, Figure 4c). The probability of observing bees also marginally decreased with increasing tree abundance (*β *= −0.208, SE = 0.107, *p* = .052). The likelihood of observing bees was higher in the wet season compared with dry season (*β* = 2.521, SE = 1.075, *p* = .019, as shown in Figure 4d). An increase in wind speed reduced the probability of finding bees (*β *= −0.913, SE = 0.526, *p* = .049).

The best model describing factors influencing Shannon diversity for bees retained only the habitat type (*β* = .175, *t* = 3.660, *p* = .0453), with fields, natural woodlots, and homesteads having higher diversities (0.175 ± 0.05, 0.171 ± 0.07, and 0.147 ± 0.06, respectively) when compared to gardens and pastures (0.068 ± 0.06 and 0.067 ± 0.06, respectively). No interaction term was significant for both the abundance and diversity models.

### 3.3 Bee community composition in relation to environmental factors and habitat type

**FIGURE 5:**
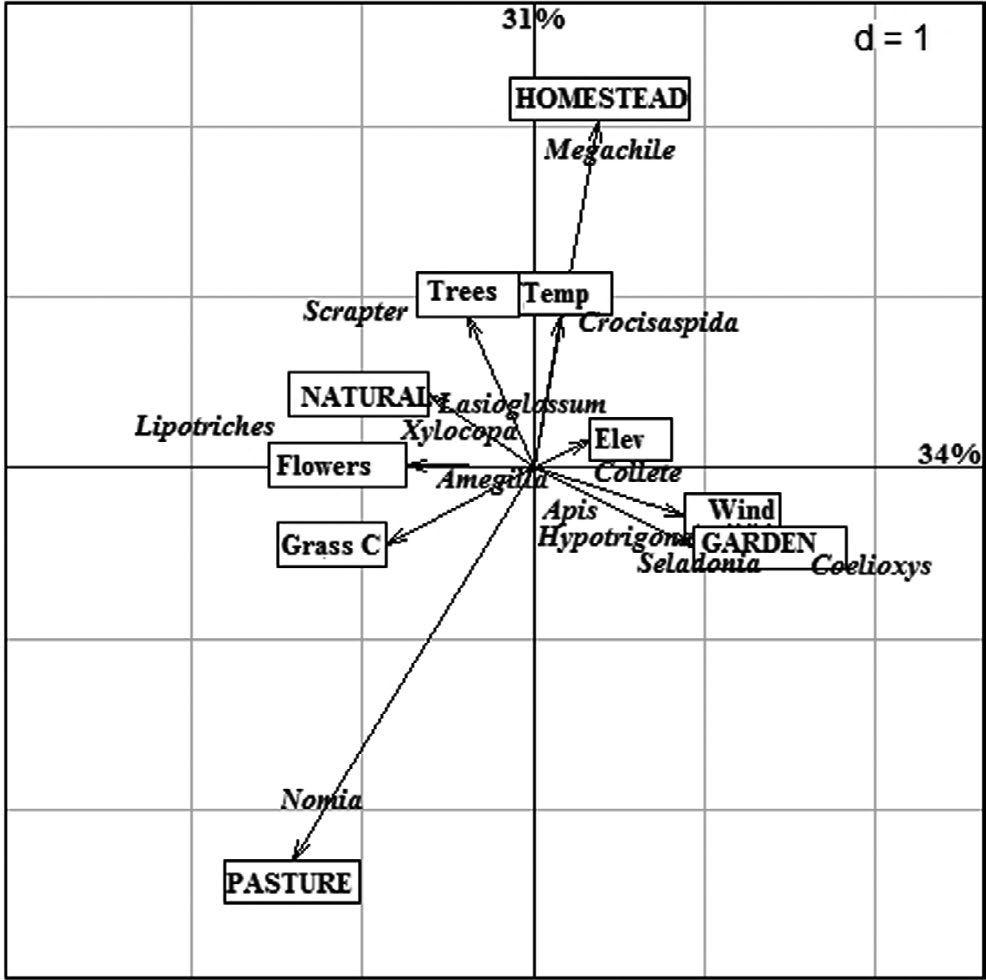
Principal component analysis triplot showing bee genus group association with environmental factors and habitat types recorded in seven sampling sites across Zvimba District in Chinhoyi Zimbabwe (August 2019–January 2020). Habitat types are denoted by (L). Key: Temp—temperature, Elev—elevation, Grass C—grass cover, Wind—wind speed, flowers—number of plant species in flower. Habitat types are in capital letters, and species are italicized

The PCA shows that axis 1 and axis 2 explained 34% and 31% of the variation, respectively, (Figure 5). Temperature was positively associated with the first axis, while number of plant species in flower was negatively associated with the first axis. *Megachile* was positively affected by temperature and was mainly associated with homesteads. *Megachile* was more associated with homesteads, while *Scrapter* with trees. *Crocisaspidia* could tolerate high temperatures. *Apis, Hypotrigona,* and *Colletes* were not negatively affected by wind speeds while *Lipotriches* favored low wind speeds and mostly found in natural woodlots. *Seladonia* and *Coelioxys* had close associations with gardens. *Xylocopa* genus was more associated with higher number of plant species in flower and more associated with natural woodlots. *Lasioglossum* was mainly found in natural woodlots, whereas *Nomia* was mostly in pastures. *Amegilla* was not influenced by any of the variables (temperature, wind speed, number of plant species in flower, tree abundance, and grass cover).

## 4 DISCUSSION

We present evidence for the effects of environmental and habitat‐related factors on bee abundance and diversity in Zvimba District rural area of Zimbabwe. Genus *Apis* was the most dominant species in the study area, and this can be attributed to its broad altitudinal and geographic ranges (De Palma et al., 2016; Gonzalez & Engel, 2004), high reproductive capacity, and added advantage of some species being domesticated in comparison with other genera. Most *Apis* species are generalist hence least affected by environmental disturbance and can persist in simplified areas unfavorable for other bees (Giannini et al., 2015; Magrach et al., 2017).

The number of plant species in flower significantly increased bee abundance confirming findings by Abrahamczyk et al. (2011), Plascencia and Philpott (2017) and Rader et al. (2011) that there is a positive relationship between bees and floral resources, regardless of latitude. The strong positive relationship between forage and bees shows that bee conservation efforts should prioritize provisioning of flowers to improve bee populations. Habitats have to be managed in ways that improve floral resources for bees. However, the probability of finding bees decreased with an increase in number of plant species in flower and also marginally decreased with an increase in number of trees. A possible explanation for these trends is that the increase in plant species in flower and tree abundance potentially has a dilution effect, as there will be increased forage choices for bees. This probably implies that the bees become more scattered in the surrounding, hence lowering the probability of encountering them within the 1‐m‐wide transect belt that was used. It is also worthy to note that the highest records of plant species in flower and tree abundance were recorded in natural woodlots; thus, other methods of capturing bees should be employed to enhance bee detection.

Provision of suitable abiotic conditions also proved important for bees with temperature significantly increasing bee abundance within a specific narrow temperature range of 20–28.5°C beyond which bee abundance declined. These findings corroborate with other studies, which have found temperature to strongly affecting foraging activity of bees (Schweiger et al., 2019) and the performance of vast majority of activities (Silva & Dean, 2000; Vollet‐Neto et al., 2011). The narrow thermal niche of bees validates reports on the potential declines of bees due to projected changes in climate, hence the need for farmers to consider different methods of regulating temperature in their farms to enhance bee activity and diversity. For example, trees due to shade and their role of evaporation and transpiration in reducing sensible heat (Hesslerová et al., 2013; Pokorný et al., 2010) can also be grown to create localized climates for protecting native bees from adverse temperature changes. There is also an urgent need for further studies on beehive designs that can provide sufficient insulation for the periods of extreme hot and cold temperatures (Greco et al., 2010; He et al., 2020) in domesticated bee species. *Megachile* and *Crocisaspidia* genera were, however, the only genera shown to be more tolerant to high temperatures according to the outcome of the PCA, highlighting the differential effects climate change will have on species and the need for species focused conservation efforts in some instances. In the endeavor to adjust hot temperatures in landscapes, strategies should ensure habitats accommodate hot‐tolerant species, otherwise conservation efforts may become perilous to some species and contradict their purpose.

Contrary to our hypothesis, bee diversity was not influenced by temperature and one possible explanation could be that species were adapted to local climatic conditions, which however may imply that temperature changes might significantly affect bee activities and ultimately survival. This result also emphasizes the differential species survival and adaptation ability in different climates; hence, knowledge of species adaptation is key in conservation efforts in different climatic zones. Management efforts can also be streamlined in specific areas to offer preferred conditions for specific adaptable species in that area (zonal conservation) (Marta‐Pedroso et al., 2007), making it more cost‐effective and impactful.

The probability of finding bees marginally decreased with increases in wind speeds, which is understandable as many findings have shown the negative impact of wind speed on flight performance (Combes & Dudley, 2009) and landing of bees (Chang et al., 2016) with implications on energy costs. Due to predicted increases in wind speeds associated with deforestation and climate change (Walker & Crane, 2000), new technologies for minimizing the impacts of strong winds on bees are imperative such as placing hives in sheltered locations (Hennessy et al., 2020) for domesticated species and windbreaks as suggested by Moisan‐DeSerres et al. (2015) to safeguard native bee species that cannot tolerate high wind speeds such as the *Lipotriches,* which were more associated with low wind speeds. Different tolerance levels of bee species to wind speeds are mainly due to diverse body sizes and morphologies (Combes & Dudley, 2009). There is also, however, the possibility of high wind speeds negatively influencing detectability of the bees, hence low abundance further underscoring the importance of using multiple bee capture methods to improve sampling.

In line with this study's hypothesis, the probability of finding bees was also significantly influenced by seasonality, with higher probability of finding bees in wet season compared with dry season. These findings concur with previous studies (Williams & Middleton, 2008) that dry seasons have scarce forage resources that limit species populations. Some species were only observed during either dry or wet season, and this could be explained by seasonality in some solitary bee species (Bosch & Vicens, 2006) or lack of appropriate floral resources at the sites during particular seasons (Wojcik et al., 2008). There is also a possibility of rare species being missed in some seasons due to difficulties in detecting them. There is therefore need to combine sampling techniques such as pan traps (Munyuli, 2013) to increase capture.

Contrary to the study's hypothesis, bee abundance was highest in homesteads as compared to natural woodlots; however, *Apis* exhibited a marked dominance over other genera in these habitat types, a situation often associated with disturbed habitats was a few tolerant species thrive. Fruit trees, landscaping with beautifying flower plants, and backyard horticultural plots in human habitations may also explain this abundance as they are important sources of forage and nesting resources for bees (Ulyshen et al., 2010), and constructions further offer suitable sites for nest thermoregulation (Cely‐Santos & Philpott, 2019). *Megachile* species, for example, was associated with homestead (Figure 5), and this can be attributed to their nesting behavior on pre‐existing man‐made cavities (Sheffield, 2017). Human habitations can therefore be targeted for bee conservation, hence further validating recommendations by Tarakini et al. (2020) for the need for bee awareness programs to reduce fear towards bees if successful conservation around homesteads is to be achieved.

Also contrary to the study's hypothesis, diversity was highest in fields followed by natural woodlots, homesteads, pastures, and gardens corroborating reports from other studies, which found fields hosting similar bee diversity to other habitat types such as meadows (Todd et al., 2016). Mass flowering of crops may be more attractive and adequate to host diverse bee species with minimal competition (Grab et al., 2017). The polyculture system of intercropping maize with crops such as butternut, beans, okra, and sweet potatoes may also have created diverse forage for bees, which attracted diverse species of bees (Vides‐Borrell et al., 2019). However, the forage resource is for short duration (wet season only, and in some instances, the flowering period does not last more than a month for all the crops), which might explain the overall observed low bee abundance in the fields. Farmers may therefore consider growing drought‐resistant herbs and flowers during off‐growing seasons to save as forage for bees.

Species assemblages also differed across habitat types highlighting the differing needs of bee species in terms of forage, nesting sites, among others. *Scrapter* genus was specifically found to be associated with natural forests and trees. According to studies by Rozen Jr and Michener (1968), the *Scrapter* genus nests in the soil, and the possible explanation for its association with natural forests could be due to minimal soil disturbance in these habitats in comparison with other habitat types (Main et al., 2019). Also, it could be possible that the genus preferred forage was in the natural forests confirming previous studies by Gess and Gess (2014) who noted the genus to have strong forage preferences for specific plant species. This finding highlights its vulnerability in the face of land‐use change and the importance of setting aside natural sites across diverse habitat types to act as refuge sites for such species.

The following genera were associated with gardens: *Seladonia*, *Coelioxys*, *Hypotrigona,* and *Apis*. The case of *Seladonia* and *Apis’ a*ssociation with gardens can be explained by the polylectic nature (Lopatin & Tregub, 2003) of most species in these genera and preference for aggregated flowers, which are normally found in gardens (Plascencia & Philpott, 2017). As such bee diversity was found to be lower in gardens and pastures in comparison with fields, natural woodlots, and homesteads, which were associated with *Lasioglossum*, *Lipotriches,* and *Xylocopa* genera, suggesting that some species were lost or driven away due to land‐use changes, thus further emphasizing the need to conserve natural habitats as refuge sites for bees. For example, *Xylocopa* genus association with natural woodlots may be driven by the need for trees (Table 2) as majority nests in wood (Hurd, 1958) meaning that any loss of wood in other land‐use types might be negatively impacting the genus.

## 5 CONCLUSION

Environmental and habitat‐related factors influence bee abundance and diversity. This study reported higher bee diversities in fields, natural woodlots, and homesteads, while greater abundances were observed in homesteads and gardens. It seems that some bee genera, through biotic homogenization, thrived better under particular conditions (*Seladonia* in gardens), while others did not. It is important to use other bee trapping methods across all habitats to increase ability to detect bees, more so in areas with many trees and plant species in flower. Results from this study, however, highlight the importance of maintaining natural habitats for bees across diverse land‐use systems as refuge sites for susceptible species. The management implications of this study are probably threefold. Firstly, an increase in the floral abundance is important in sustaining high bee populations, but for greater bee diversity, the number of plant species in flower should also be increased. Secondly, fields could be managed to offer forage for bees during the dry season by encouraging or planting fast‐growing herbs and shrubs (even in the contour lines and field edges). Thirdly, there is a need for farmers to consider methods of regulating temperature and wind speeds (i.e., windbreaks and beehive shelters for nest thermoregulation) to cushion bees from extreme weather elements.

## CONFLICT OF INTEREST

The author(s) declare(s) that there is no conflict of interest.

## AUTHORS CONTRIBUTIONS

GT, RM, AC: Experimental design. GT, TT: Experiment; analysis of data with input from AC. GT: Writing of the manuscript with extensive input from RM, AC, and TT.

## Supporting information

Supplementary Material

## Data Availability

Data were deposited in the Dryad data repository https://doi.org/10.5061/dryad.9cnp5hqhh.
