# Supplementary material for: Drivers of diversity and community structure of bees in an agroecological region of Zimbabwe
Source: Ecol Evol. 2021 May 1;11(11):6415–26. doi: 10.1002/ece3.7492 (PMC8207386; doi:10.1002/ece3.7492)
Supplement: Supplementary file 1 — Supplementary Material [file ECE3-11-6415-s001.docx]

Appendix 1: Results on correlations of categories of variables related to A) weather elements, B) woody vegetation, C) Herbaceous vegetation and D) flowers. The asterix denotes level of significance, with codes *P*<0.0001‘***’, 0.01<*P*<0.001‘**’ and *P*<0.01‘*’.

| Category |  | Temp | Humidity | Ground cover | Tree abundance | Grass cover | No.PlantsInFlower |
| --- | --- | --- | --- | --- | --- | --- | --- |
| A | Hum | -0.499*** | 1 |  |  |  |  |
|  | Light Intensity | 0.475** | -0.444** |  |  |  |  |
| B | Tree abundance | N/A | N/A | -0.293* | 1 |  |  |
|  | No.PlantsInFlower | N/A | N/A | -0.325** | 0.903*** |  |  |
| C | Grass height | N/A | N/A | N/A | N/A | 0.436*** |  |
| D | Flower abundance | N/A | N/A | N/A | N/A | N/A | 0.408** |
